# Interface-Controlled GO–CoFe_2_O_4_–Silicone Nanocomposite with Magnetic and Adsorptive Functionality

**DOI:** 10.3390/nano16060345

**Published:** 2026-03-11

**Authors:** Rabiga M. Kudaibergenova, Aitekova R. Anar, Gulzat K. Demeuova, Nazgul S. Murzakasymova, Marzhan S. Kalmakhanova, Seitzhan A. Orynbayev, Helder T. Gomes, Gulnar K. Sugurbekova

**Affiliations:** 1 Department of Chemistry and Chemical Technology, Faculty of Technology, M. Kh. Dulaty Taraz University, 60 Tole Bi Street, Taraz 080000, Kazakhstan; rm.kudajbergenova@dulaty.kz (R.M.K.); aaytekova89@mail.ru (A.R.A.); ms.kalmakhanova@dulaty.kz (M.S.K.); sa.orynbayev@dulaty.kz (S.A.O.); 2 School of Engineering and Digital Sciences, Nazarbayev University, Astana 010000, Kazakhstan; gdemeuova@nu.edu.kz; 3 Centro de Investigação de Montanha (CIMO), LA SusTEC, Instituto Politécnico de Bragança, Campus de Santa Apolónia, 5300-253 Bragança, Portugal; htgomes@ipb.pt; 4 Department of Chemistry, Faculty of Natural Sciences, L. N. Gumilyov Eurasian National University, Astana 010000, Kazakhstan

**Keywords:** graphene oxide, cobalt ferrite, magnetic sponge, oil/water separation, adsorption

## Abstract

The development of interface-engineered, multifunctional nanostructured materials with controllable surface and magnetic properties remains a critical challenge in wastewater treatment and environmental remediation. In this work, a novel GO–CoFe_2_O_4_–Silicone Magnetic Sponge was successfully fabricated through the integration of graphene oxide and CoFe_2_O_4_ magnetic nanoparticles within a silicone-modified porous sponge matrix. The resulting material combines superhydrophobicity, oleophilicity, high adsorption capacity, and magnetic responsiveness in a single architecture. The prepared sponge exhibited a high water contact angle of 161.5°, confirming its superhydrophobic nature, while maintaining excellent structural integrity during repeated use. Vibrating sample magnetometry revealed clear ferrimagnetic behavior, enabling rapid magnetic manipulation and efficient recovery of the sponge from aqueous media. The GO–CoFe_2_O_4_–Silicone Magnetic Sponge demonstrated strong adsorption performance toward a wide range of oils and organic solvents, including chloroform, olive oil, toluene, ethanol, acetone, gasoline, and hexane, with adsorption capacities remaining stable over multiple cycles. Furthermore, the sponge showed outstanding separation efficiency exceeding 98.3% for various oil/water and organic solvent/water mixtures, both in batch and continuous vacuum-assisted separation systems. The adsorption capacity and separation efficiency were retained after repeated adsorption–desorption cycles, indicating excellent reusability and durability. Owing to its synergistic combination of surface chemistry, porous structure, and magnetic functionality, the GO–CoFe_2_O_4_–Silicone Magnetic Sponge represents a promising candidate for practical applications in oil spill cleanup and wastewater treatment.

## 1. Introduction

The contamination of aquatic environments by oil and oily wastewaters remains a critical environmental challenge due to frequent oil spills, industrial discharge, and the increasing complexity of emulsified oil–water mixtures [1,2]. Conventional separation techniques such as gravity separation, skimming, and coagulation often suffer from low efficiency, high energy consumption, and secondary pollution, which significantly limit their large-scale application in the treatment of complex wastewater streams. Therefore, the development of advanced separation materials with high selectivity, efficient recovery capability, and long-term reusability has become an important research focus for sustainable oily wastewater remediation.

Graphene oxide (GO), a two-dimensional carbonaceous material with abundant oxygen-containing functional groups and a high specific surface area, has emerged as a promising candidate for oil–water separation and demulsification applications. GO nanosheets can interact strongly with oil components through π–π interactions, hydrophobic forces, and electrostatic attraction, thereby enhancing separation efficiency in both free oil and emulsified systems [3]. However, pristine GO is inherently hydrophilic and lacks an efficient recovery mechanism after use, which restricts its practical applicability in water treatment systems [4]. To overcome these limitations, GO has frequently been combined with magnetic nanoparticles (MNPs), enabling rapid magnetic separation of the sorbent after treatment without extensive filtration or centrifugation [5]. For instance, magnetic GO systems have demonstrated remarkable demulsification performance, achieving separation efficiencies approaching 99.98% with excellent recyclability under an external magnetic field [6]. Recent studies have also reported magnetically recoverable GO-based demulsifiers with tunable surface chemistry capable of maintaining high separation efficiency across a wide range of environmental conditions [7]. In addition, novel magnetic Janus graphene oxide systems have been developed for efficient and recyclable demulsification of crude oil-in-water emulsions, demonstrating enhanced separation performance and operational stability [8].

Beyond magnetic modification, tailoring surface wettability plays a crucial role in selective oil–water separation. Hydrophobic and oleophilic surfaces preferentially interact with oil phases while repelling water, thereby significantly enhancing separation performance. In membrane-based systems, incorporation of GO into polymer matrices has been reported to improve permeability, antifouling behavior, and separation selectivity in oil–water emulsion filtration [9]. In recent years, three-dimensional porous sponge materials have also attracted increasing attention as efficient sorbents due to their high porosity, low density, and large adsorption capacity. Advanced polymeric sponge architectures have been demonstrated as promising materials for environmental remediation and marine ecosystem protection owing to their excellent absorption capacity and mechanical robustness [10]. Similarly, functionalized sponge systems incorporating porous frameworks and surface-engineered coatings have been reported to achieve efficient oily wastewater separation through synergistic capillary absorption and selective wettability mechanisms [11].

Magnetic modification of sponge-based sorbents further enhances their practical applicability by enabling convenient magnetic manipulation and recovery. Cobalt ferrite (CoFe_2_O_4_) is a particularly attractive magnetic material due to its high coercivity, chemical stability, and tunable magnetic properties [12]. When combined with graphene-based materials, CoFe_2_O_4_ nanoparticles can enhance magnetic recovery while maintaining strong interfacial interactions with contaminants. In addition, magnetic nanocomposite adsorbents incorporating polymer matrices and magnetic nanoparticles have shown excellent performance in removing various contaminants from aqueous media, highlighting the versatility of magnetic hybrid materials in environmental applications [13].

Although magnetic oil–water separation systems have been widely investigated, most existing studies focus primarily on single-function enhancement, such as magnetic recovery or surface wettability modification alone. Systematic integration of interfacial chemistry, magnetic responsiveness, and selective wettability control within a unified hierarchical porous architecture remains relatively limited. In particular, rational interface-engineered designs that simultaneously optimize dispersion stability, interfacial bonding, pore accessibility, magnetic recyclability, and surface-energy regulation are still scarce.

In this work, we propose a synergistic GO–CoFe_2_O_4_–silicone magnetic sponge system, where each component performs a distinct and complementary function:

(i) GO acts as an interfacial scaffold providing active bonding sites, surface roughness, and adsorption capability;
(ii) CoFe_2_O_4_ nanoparticles impart strong magnetic responsiveness and enable rapid, contactless recovery;
(iii) Silicone modification reduces surface energy and induces hydrophobicity and oleophilicity for selective oil capture.

This interface-engineered architecture enables the simultaneous realization of selective wettability, hierarchical porosity, efficient magnetic recovery, and structural stability, resulting in a multifunctional separation platform. The novelty of this work lies in the synergistic coupling of interfacial chemistry, magnetic functionality, and wettability engineering within a three-dimensional porous sponge structure, providing a scalable, recyclable, and high-performance material for oil–water separation and wastewater treatment applications.

## 2. Materials and Methods

### 2.1. Chemicals and Apparatus

Silicone oil AS 100 (viscosity ≈ 100 mPa·s at 25 °C) and hexane (≥95%) were purchased from Sigma-Aldrich (St. Louis, MO, USA). Polyurethane sponge (PU, density 22 kg/m^3^) was obtained from a local commercial supplier (Taraz, Kazakhstan). Natural graphite flakes (carbon content ≥99%), sodium nitrate (NaNO_3_), sulfuric acid (H_2_SO_4_), potassium permanganate (KMnO_4_), cobalt nitrate hexahydrate (Co(NO_3_)_2_·6H_2_O), iron(III) nitrate nonahydrate (Fe(NO_3_)_3_·9H_2_O), acetic acid, ethylene glycol, and 2-methoxyethanol were of analytical grade and used as received. Deionized water (18.2 MΩ·cm) produced by a Milli-Q purification system (Millipore, Burlington, MA, USA) was used throughout all experiments.

The structural and physicochemical properties of graphite, graphene oxide, and the resulting GO–CoFe_2_O_4_–silicone composite were characterized using complementary analytical techniques. Raman spectroscopy measurements were performed using a LabRAM HR Evolution Raman spectrometer (Horiba Scientific, Kyoto, Japan) equipped with a 532 nm laser to evaluate structural order and defect characteristics. Fourier-transform infrared (FTIR) spectra were recorded on a Nicolet iS5 FTIR spectrometer (Thermo Fisher Scientific, Waltham, MA, USA) to identify surface functional groups and chemical bonding. Phase composition and crystallinity were analyzed by X-ray diffraction (XRD) using a SmartLab diffractometer (Rigaku Corporation, Tokyo, Japan). Surface morphology and microstructural features were examined by scanning electron microscopy (SEM) using a Crossbeam 540 microscope (Carl Zeiss AG, Oberkochen, Germany). Wettability properties were evaluated through static water contact angle measurements using an OCA 15EC contact angle system (DataPhysics Instruments GmbH, Filderstadt, Germany). The contact angle values were determined using SCA 20 software (DataPhysics Instruments GmbH, Filderstadt, Germany).

### 2.2. Preparation of Graphene Oxide (GO)

GO was synthesized from natural graphite flakes using a modified Hummers method [14]. In a typical procedure, sodium nitrate (0.5 g) was dissolved in concentrated sulfuric acid (23 mL, 95 wt%) under continuous stirring at room temperature. The mixture was subsequently cooled in an ice bath, and graphite flakes (1 g) were gradually introduced under controlled stirring conditions. Potassium permanganate (3 g) was added portionwise at regular intervals to prevent excessive temperature rise and ensure controlled oxidation. During this step, the reaction temperature was carefully maintained below 15 °C. After complete addition of the oxidant, the reaction mixture was slowly heated to 35 °C and maintained under vigorous stirring for 24 h to promote effective intercalation and oxidation of graphite layers. Subsequently, distilled water was added dropwise to dilute the mixture, while the temperature was kept below 70 °C to avoid violent reactions. Further dilution with distilled water was performed after cooling, followed by gradual addition of hydrogen peroxide solution (34.5 wt%) until the suspension turned bright yellow, indicating the completion of oxidation and reduction in residual permanganate species. The resulting suspension was repeatedly washed with aqueous hydrochloric acid solution (10 wt%) and deionized water to remove metal ions and acidic residues. The purified GO dispersion was then subjected to ultrasonic treatment for 2 h to facilitate exfoliation of graphite oxide into graphene oxide sheets. The exfoliated product was collected by centrifugation at 3500 rpm for 40 min, and the obtained GO precipitate was dried at 120 °C to obtain graphene oxide powder.

### 2.3. Synthesis of Cobalt Ferrite (CoFe_2_O_4_) Nanoparticles

**Figure 1:**
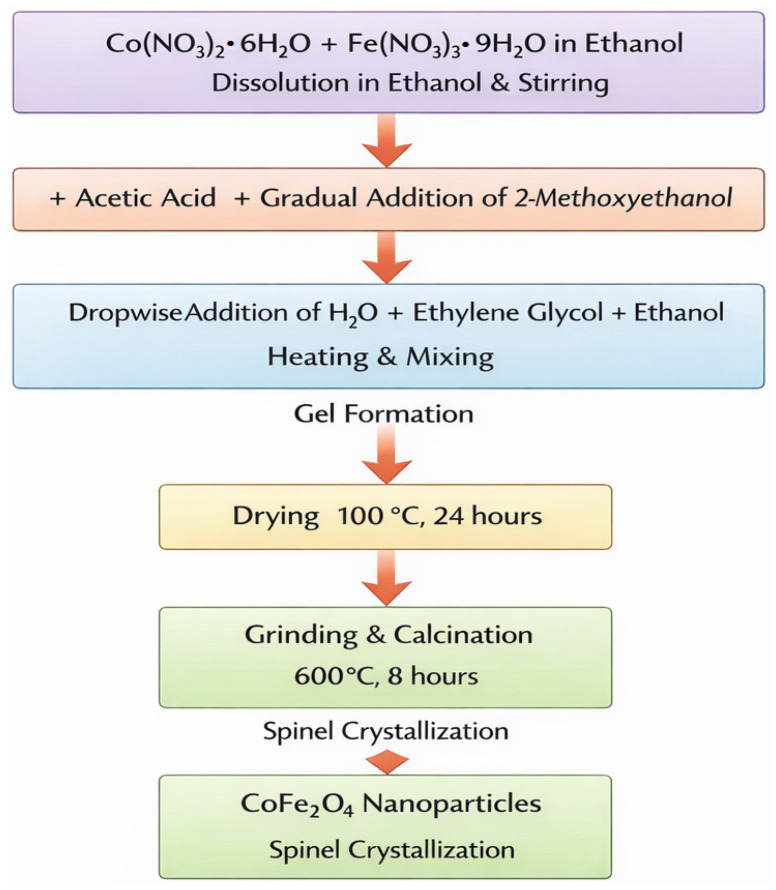
Synthesis of CoFe_2_O_4_ Nanoparticles.

CoFe_2_O_4_ nanoparticles were synthesized via a sol–gel approach using metal nitrate precursors [12]. Stoichiometric amounts of cobalt(II) nitrate hexahydrate and iron(III) nitrate nonahydrate were dissolved in ethanol under continuous magnetic stirring to obtain a homogeneous precursor solution. The mixture was maintained in a three-necked flask equipped with a condenser to ensure controlled reaction conditions. To promote hydrolysis and gel formation, acetic acid (1 mL) was introduced as a catalyst, followed by the gradual addition of 2-methoxyethanol as a co-solvent. Subsequently, a mixed solution of deionized water, ethylene glycol, and ethanol was added dropwise under heating and stirring, leading to the formation of a viscous gel through progressive polycondensation reactions. The resulting gel was dried in an oven at 100 °C for 24 h to remove residual solvents and organic components. The dried precursor was gently ground to obtain a fine powder and then calcined in a muffle furnace at 600 °C for 8 h. This thermal treatment facilitated the crystallization of the spinel CoFe_2_O_4_ phase and removal of remaining organic residues, yielding cobalt ferrite nanoparticles with well-defined magnetic properties (Figure 1).

### 2.4. Preparation of GO–CoFe_2_O_4_–Silicone Magnetic Sponge

A three-dimensional magnetic sponge was fabricated using a polyurethane (PU) sponge as a porous scaffold. The PU sponge was first ultrasonically cleaned in ethanol and deionized water for 10 min each, followed by drying at 60 °C for 5 h to remove surface contaminants and residual solvents. Subsequently, the dried PU sponge was immersed in an aqueous graphene oxide (GO) dispersion (2 mg mL^−1^) and gently compressed repeatedly to ensure complete infiltration of GO sheets into the interconnected pore network. The impregnation process was carried out for 30 min under sonication to promote uniform penetration and adhesion of GO within the porous structure. After impregnation, the sponge was dried at 60 °C for 3 h to promote stable attachment of GO onto the PU skeleton. CoFe_2_O_4_ nanoparticles were then introduced by immersing the GO-coated sponge into an aqueous suspension of cobalt ferrite nanoparticles (5 mg mL^−1^). The composite was maintained under gentle stirring for 1 h, followed by ultrasonic treatment for 15 min to facilitate uniform deposition of magnetic nanoparticles onto the GO-modified pore surfaces through interfacial interactions. The sponge was then removed and dried at 60 °C for 3 h to stabilize the GO–CoFe_2_O_4_ structure and impart magnetic responsiveness. To render the sponge hydrophobic and oleophilic, silicone oil AS 100 was dissolved in hexane to form a dilute solution (5 wt%). The GO–CoFe_2_O_4_-loaded sponge was immersed in this solution for 20 min, followed by mild ultrasonication for 2 h to ensure uniform coating of the internal pore surfaces. The sponge was then removed and dried at 60 °C for 3 h, followed by drying under ambient conditions for 12 h to ensure complete solvent evaporation. This treatment resulted in the formation of a thin silicone coating on the internal pore surfaces without blocking pore connectivity. The obtained material was denoted as the GO–CoFe_2_O_4_–silicone sponge.

## 3. Results and Discussion

The interfacial coupling between graphene oxide (GO) and CoFe_2_O_4_ nanoparticles is critical for ensuring structural robustness, magnetic responsiveness, and adsorption functionality of the composite system. The immobilization of CoFe_2_O_4_ onto GO is governed by coordination interactions between surface metal cations (Co^2+^/Fe^3+^) and oxygen-containing functional groups of GO, supplemented by electrostatic attraction and hydrogen bonding. These interactions enable homogeneous nanoparticle anchoring while suppressing agglomeration within the three-dimensional porous scaffold.

### 3.1. FTIR and Raman Analysis of Graphene Oxide

**Figure 2:**
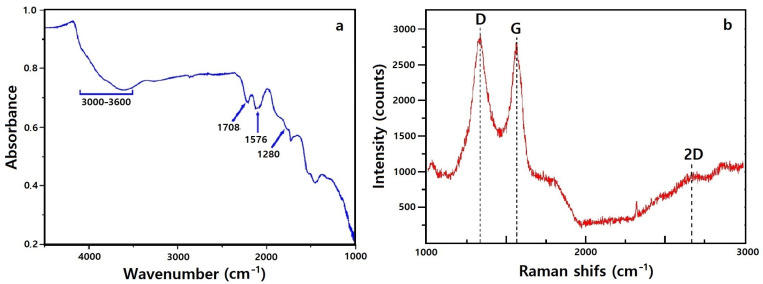
(**a**) FTIR spectrum and (**b**) Raman spectrum of synthesized GO.

FTIR spectroscopy was employed to confirm the successful oxidation of graphite and the formation of oxygen-functionalized GO (Figure 2a). A broad absorption band at 3000–3600 cm^−1^ corresponds to O–H stretching vibrations from hydroxyl groups and adsorbed moisture. Characteristic peaks at 1708, 1576, and 1280 cm^−1^ are assigned to C=O stretching (carbonyl/carboxyl groups), C=C skeletal vibrations of sp^2^ domains, and C–O–C stretching of epoxy groups, respectively [15]. The presence of these functionalities confirms effective oxidative exfoliation and provides chemically reactive sites for subsequent interfacial interactions.

These oxygenated groups facilitate coordination bonding with surface Co^2+^ and Fe^3+^ ions of CoFe_2_O_4_, promoting stable nanoparticle immobilization on GO sheets. Such interfacial interactions are essential for maintaining dispersion stability and ensuring uniform distribution of magnetic domains throughout the porous network.

The silicone modification is integrated onto the GO–CoFe_2_O_4_ framework predominantly through non-covalent interactions. Hydrogen bonding and dipole interactions between silicone chains and oxygenated GO surfaces, combined with van der Waals interactions at the nanoparticle interface, result in a conformal low-surface-energy coating. Importantly, this interface-controlled assembly preserves pore accessibility while imparting durable hydrophobic and oleophilic characteristics necessary for selective oil–water separation.

Raman spectroscopy further verifies the structural characteristics of GO (Figure 2b). The D band (~1348 cm^−1^) is associated with disorder-induced breathing modes of sp^2^ carbon rings, whereas the G band (~1590 cm^−1^) corresponds to in-plane vibrations of sp^2^ carbon atoms [16]. The observed ID/IG ratio of ≈0.85 indicates significant defect generation and lattice distortion resulting from oxidation. Such defect-rich domains enhance chemical reactivity and provide anchoring sites for nanoparticle attachment, supporting effective composite formation.

Collectively, the FTIR and Raman results confirm the successful synthesis of defect-rich, oxygen-functionalized GO suitable for interfacial coupling with magnetic nanoparticles.

### 3.2. XRD Analysis of Graphene Oxide and CoFe_2_O_4_ Nanoparticles

**Figure 3:**
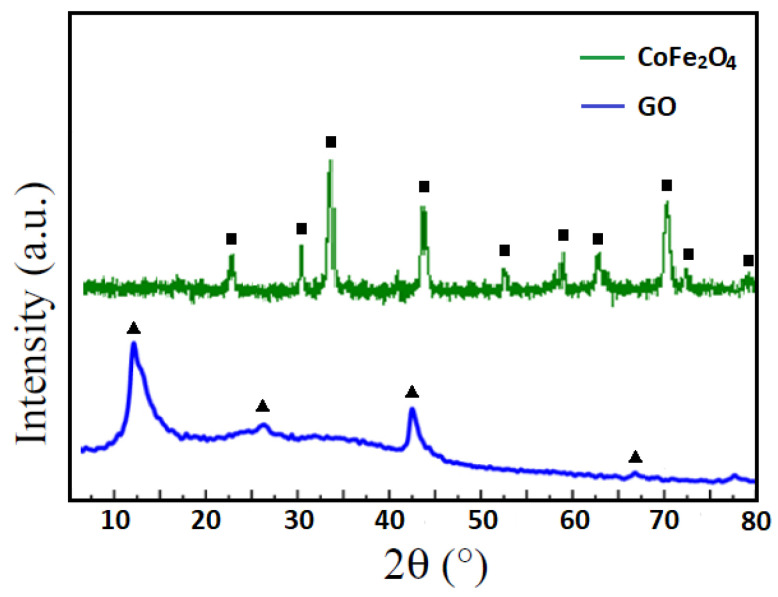
XRD Analysis of Graphene Oxide and CoFe_2_O_4_ Nanoparticles.

X-ray diffraction (XRD) was used to investigate the crystalline structure of the synthesized graphene oxide (GO) and cobalt ferrite (CoFe_2_O_4_) nanoparticles. The results obtained are presented in Figure 3. The XRD pattern of GO exhibits a broad peak around 2θ ≈ 11°, corresponding to the (001) plane, which is characteristic of the expanded interlayer spacing caused by the introduction of oxygen-containing functional groups during the oxidation of graphite (Hummers method) [17]. A weaker, broader peak around 2θ ≈ 26° indicates the presence of residual graphitic domains within the GO sheets.

The XRD pattern of CoFe_2_O_4_ nanoparticles shows distinct diffraction peaks at 2θ ≈ 30.1°, 35.5°, 43.1°, 53.5°, 57.0°, and 62.6°, corresponding to the (220), (311), (400), (422), (511), and (440) planes, respectively, which are in agreement with the standard spinel structure of cubic cobalt ferrite (JCPDS No. 22-1086) [18]. The sharpness and intensity of these peaks indicate good crystallinity of the CoFe_2_O_4_ nanoparticles.

These results confirm the successful synthesis of graphene oxide and crystalline CoFe_2_O_4_ nanoparticles used for the preparation of the composite sponge. Since the composite fabrication involves physical immobilization of pre-synthesized GO sheets and CoFe_2_O_4_ nanoparticles onto the polyurethane scaffold without additional calcination, the crystalline spinel structure of CoFe_2_O_4_ is expected to remain preserved in the final GO–CoFe_2_O_4_–silicone composite. Such an interface-driven assembly allows the magnetic nanoparticles to be uniformly anchored within the porous structure while maintaining their intrinsic magnetic properties and the functional surface chemistry of GO.

### 3.3. Morphological and Elemental Analysis (SEM–EDS)

The surface morphology of the synthesized GO, CoFe_2_O_4_ nanoparticles, and the final GO–CoFe_2_O_4_–Silicone Magnetic Sponge was investigated using scanning electron microscopy (SEM).

**Figure 4:**
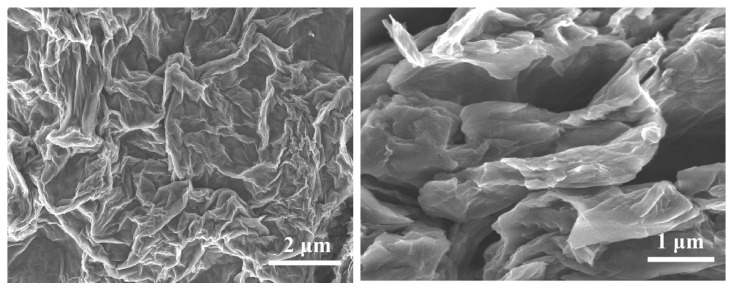
SEM images of graphene oxide (GO) nanosheets.

SEM images of GO reveal the typical two-dimensional sheet-like structure with wrinkled and layered morphology (Figure 4), characteristic of oxidized graphite [19]. The sheets appear partially exfoliated and loosely connected, reflecting the introduction of oxygen-containing functional groups, which induce distortion and prevent complete restacking of the layers. This morphology provides a high surface area and abundant reactive sites for subsequent composite formation.

**Figure 5:**
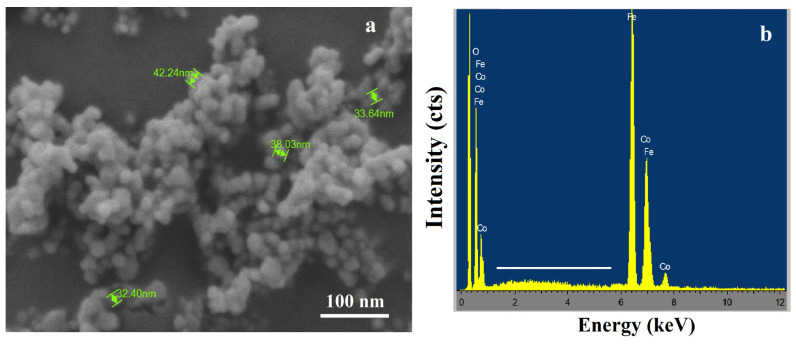
(**a**) SEM image; (**b**) EDS analysis of cobalt ferrite (CoFe_2_O_4_) nanoparticles.

Cobalt ferrite (CoFe_2_O_4_) nanoparticles display a nearly uniform distribution of quasi-spherical particles with sizes in the range of 10–50 nm (Figure 5) [20]. The images indicate well-defined spinel structures with a moderate degree of surface roughness, suggesting a controlled crystallization process during the thermal treatment. The uniform particle size and surface characteristics are conducive to homogeneous incorporation onto the sponge skeleton and facilitate magnetic responsiveness. EDS analysis confirms the presence of Co and Fe elements, providing direct compositional evidence for the formation of cobalt ferrite with the expected elemental composition.

**Figure 6:**
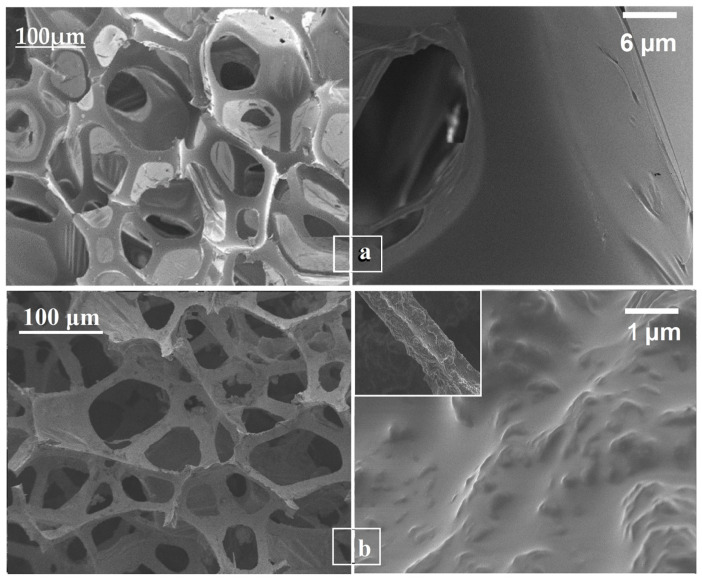
SEM images of (**a**) pristine polyurethane (PU) sponge and (**b**) GO–CoFe_2_O_4_–Silicone Magnetic Sponge after surface modification.

SEM analysis of the pristine PU sponge (Figure 6a) reveals a well-defined three-dimensional porous network with smooth pore walls and a clean polymer skeleton, serving as a reference for subsequent functionalization. Upon modification, the GO–CoFe_2_O_4_–Silicone Magnetic Sponge retains this interconnected macroporous architecture (Figure 6b) while exhibiting a significant enhancement in hierarchical micro/nano-scale surface roughness. The sponge skeleton is densely decorated with graphene oxide sheets and uniformly distributed CoFe_2_O_4_ nanoparticles, indicating strong interfacial interactions that prevent nanoparticle aggregation. This uniform distribution ensures consistent magnetic responsiveness and adsorption throughout the sponge [17]. The combination of preserved macroporosity and hierarchical surface roughness enhances superhydrophobicity and oleophilicity, enabling rapid oil uptake and efficient oil/water separation, as well as stable performance over multiple adsorption–desorption cycles.

### 3.4. VSM and Magnetic Properties

**Figure 7:**
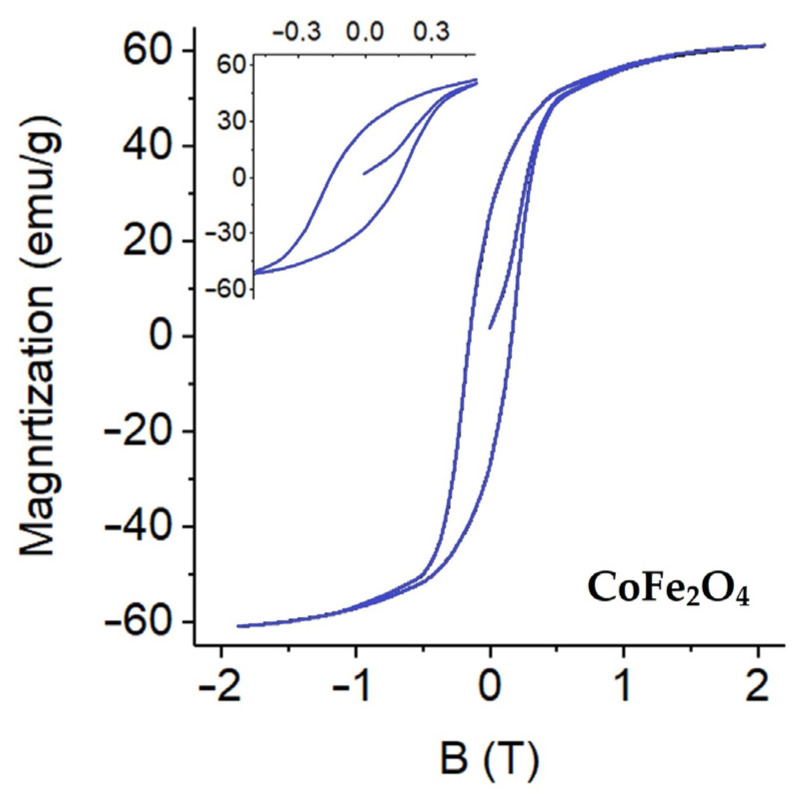
VSM hysteresis loop of CoFe_2_O_4_ nanoparticles.

The magnetic properties of the synthesized CoFe_2_O_4_ nanoparticles were evaluated using vibrating sample magnetometry (VSM), and the corresponding hysteresis loop is presented in Figure 7. The magnetization curve exhibits a clear and well-defined hysteresis behavior, confirming the ferrimagnetic nature characteristic of spinel CoFe_2_O_4_ systems [12]. The hysteresis loop shows distinct coercivity (Hc) and remanent magnetization (Mr), indicating the presence of stable magnetic domains and pronounced magnetic anisotropy. Such behavior is typical of magnetically hard ferrite materials and is advantageous for applications requiring reliable magnetic manipulation, separation, and recovery. The CoFe_2_O_4_ nanoparticles synthesized via the sol–gel method exhibit a saturation magnetization (Ms) of approximately 60–62 emu g^−1^ and a coercivity (Hc) of about 150 Oe at room temperature, which fall within the expected range for spinel cobalt ferrite prepared under similar thermal conditions. The absence of superparamagnetic behavior further confirms the formation of a well-crystallized ferrite phase rather than ultrasmall isolated particles. Since the composite fabrication involves physical immobilization of pre-synthesized CoFe_2_O_4_ nanoparticles onto the GO-modified polyurethane sponge without additional calcination, the intrinsic magnetic properties of the ferrite phase are expected to be preserved after incorporation into the composite structure. The magnetic responsiveness of the final GO–CoFe_2_O_4_–silicone sponge was experimentally verified by its rapid attraction toward an external magnet (Supplementary Video S1), confirming its practical magnetic recoverability.

The loading of the magnetic phase in the GO–CoFe_2_O_4_–silicone sponge was estimated based on the precursor suspension composition. The CoFe_2_O_4_ suspension used for impregnation had a concentration of 5 mg mL^−1^ and a total volume of 30 mL, corresponding to 150 mg of cobalt ferrite nanoparticles introduced during the deposition step. The nominal loading of the magnetic component can be expressed as

(1) $$L_{Co{Fe}_{2}O_{4}(\%)}=\frac{m_{Co{Fe}_{2}O_{4}}}{m_{composite}}\times 100$$

where $m_{Co{Fe}_{2}O_{4}}$ represents the mass of cobalt ferrite introduced during the impregnation step and $m_{composite}$ is the mass of the final dried sponge. Considering typical immobilization efficiencies for immersion coating processes, the effective CoFe_2_O_4_ loading in the composite is estimated to be approximately 20–25 wt%, which is consistent with values reported for similar magnetic sponge systems.

### 3.5. Hydrophobic Properties of the GO–CoFe_2_O_4_–Silicone Magnetic Sponge

**Figure 8:**
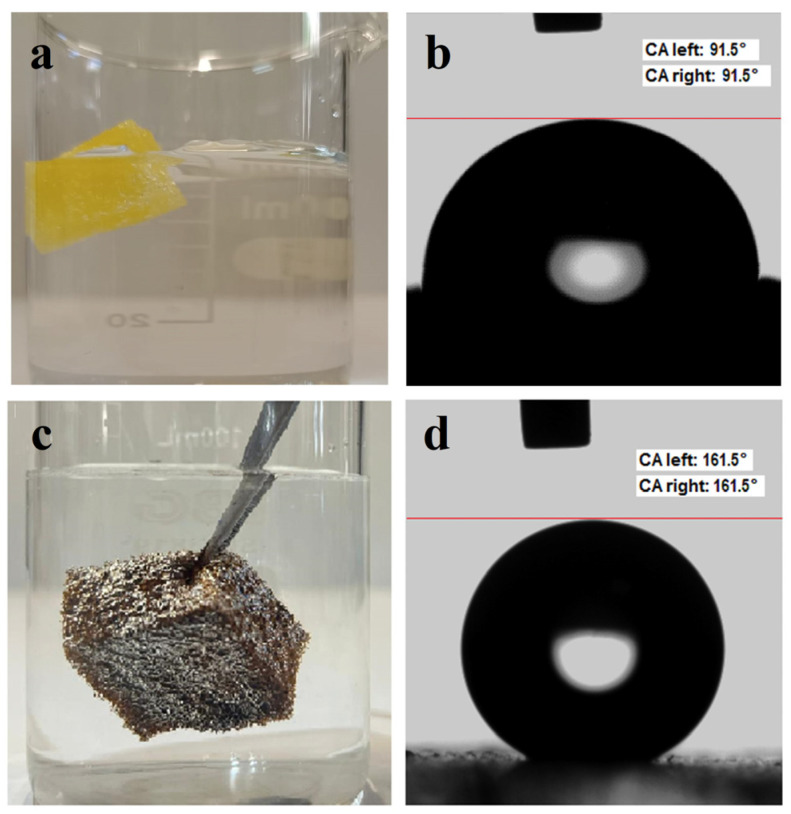
Wettability of pristine PU sponge and GO–CoFe_2_O_4_–silicone magnetic sponge: (**a**) pristine PU sponge in water; (**b**) contact angle of pristine PU sponge (≈91.5°); (**c**) water repellency of the GO–CoFe_2_O_4_–silicone magnetic sponge; (**d**) contact angle of the modified sponge (161.5°).

The hydrophobic properties of the GO–CoFe_2_O_4_–Silicone Magnetic Sponge were evaluated by visual immersion tests and static water contact angle measurements. The pristine PU sponge exhibited complete water infiltration and sank immediately upon immersion in water (Figure 8a), with a measured static water contact angle of 90.5° (Figure 8b), indicating hydrophilic–neutral surface behavior (Figure 8b). In contrast, the GO–CoFe_2_O_4_–Silicone Magnetic Sponge shows a characteristic silvery mirror-like appearance when brought into contact with water (Figure 8c), which is typically associated with the formation of a trapped air layer at the solid–liquid interface and is a clear indication of hydrophobic behavior [21]. The static water contact angle of the modified sponge surface was measured to be 161.5° (Figure 8d), confirming its superhydrophobic nature [22]. Such a high contact angle can be attributed to the synergistic effect of the low surface energy of the silicone oil coating and the hierarchical surface roughness provided by the porous sponge framework and the embedded nanostructures. The strong water repellency prevents water penetration into the sponge pores, allowing the material to remain afloat on the water surface and maintain its structural integrity during contact with aqueous media. This superhydrophobic behavior is particularly advantageous for selective oil absorption and magnetic recovery applications, as it minimizes water uptake and enhances the durability and reusability of the sponge in wastewater treatment processes.

### 3.6. Absorption and Separation Performance of the GO–CoFe_2_O_4_–Silicone Magnetic Sponge

The absorption and separation performance of the GO–CoFe_2_O_4_–Silicone Magnetic Sponge was evaluated using various oil/water and organic solvent/water mixtures, including chloroform/water, olive oil/water, acetone/water, hexane/water, toluene/water, gasoline/water, and ethanol/water systems. In a typical separation experiment, the sponge was immersed in a mixture of water and oil or organic solvent with a known composition (Figure S1). Due to its superhydrophobic and oleophilic nature, the organic phase was rapidly absorbed, while water was effectively repelled. After complete absorption of the oil or organic solvent, the sponge was removed from the mixture, and the residual amount of organic liquid remaining in the water was determined.

The separation efficiency (R, %) was calculated using Equation (2).

(2) $$R\%=\frac{m0-m1}{m0}\times 100$$

where m0 is the initial mass of oil or organic solvent in the water before separation, and m1 is the mass of oil or organic solvent remaining in the water after separation.

The GO–CoFe_2_O_4_–Silicone Magnetic Sponge exhibited excellent separation efficiencies for all tested systems. The separation efficiencies were calculated to be 98.82% for chloroform/water, 99.31% for olive oil/water, 98.74% for acetone/water, 99.01% for hexane/water, 98.69% for toluene/water, 98.58% for gasoline/water, and 99.16% for ethanol/water mixtures, respectively. These high values indicate that only trace amounts of organic liquids remained in the water after separation.

In addition, the absorption capacity of the GO–CoFe_2_O_4_–Silicone Magnetic Sponge was investigated by immersing a pre-weighed sponge into oil or organic solvents for a fixed time (20 s). After absorption, the sponge was removed and weighed immediately to minimize mass loss. The absorption capacity (Q, g/g) was calculated according to Equation (3).

(3) $$Q=\frac{m2-m1}{m1}$$

where m1 is the initial mass of the dry sponge and m2 is the mass of the sponge after absorption. Each measurement was performed three times, and the average value was used.

**Figure 9:**
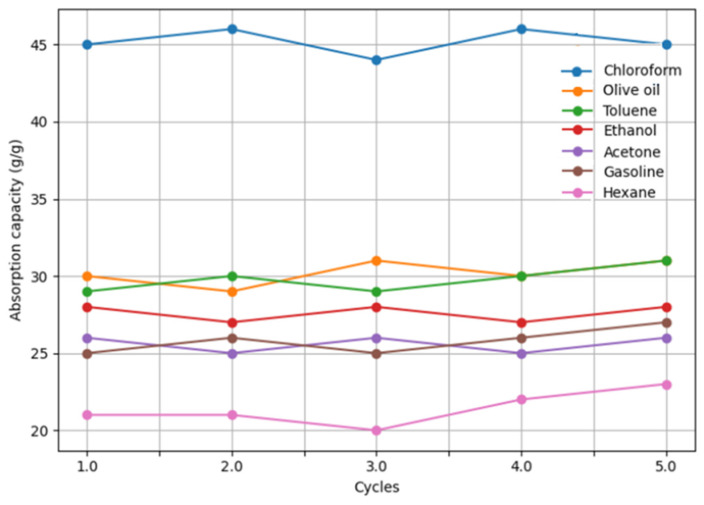
Cycling stability of absorption capacity of the GO–CoFe_2_O_4_–Silicone Magnetic Sponge for various organic liquids.

As shown in Figure 9, the sponge demonstrated high absorption capacities toward all tested liquids. The maximum absorption capacity was observed for chloroform (44.8 g/g), followed by olive oil (31.2 g/g), toluene (29.6 g/g), acetone (27.8 g/g), ethanol (28.9 g/g), gasoline (25.6 g/g), and hexane (21.4 g/g). The higher absorption capacity for chloroform can be attributed primarily to its higher density (1.49 g/cm^3^), which enhances intermolecular hydrophobic interactions between the organic liquid and the sponge surface.

The recyclability of the GO–CoFe_2_O_4_–Silicone Magnetic Sponge was evaluated over five consecutive absorption–desorption cycles (Figure 9). After each cycle, the absorbed oil or solvent was removed by simple mechanical squeezing, followed by drying. The results show that the absorption capacity remained nearly constant for all tested liquids over five cycles, with no obvious structural degradation or performance loss. This demonstrates the excellent mechanical stability and reusability of the sponge.

Overall, the GO–CoFe_2_O_4_–Silicone Magnetic Sponge exhibits high absorption capacity (21–45 g/g), excellent separation efficiency (>98.3%), and outstanding recyclability. In addition, its magnetic responsiveness enables facile manipulation and recovery from aqueous environments, making it a promising material for efficient oil spill remediation and wastewater purification [23].

### 3.7. Water Contact Angle Stability of the GO–CoFe_2_O_4_–Silicone Magnetic Sponge

**Figure 10:**
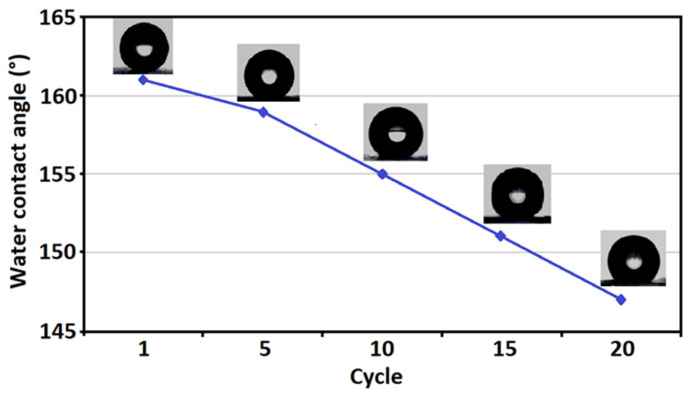
Water contact angle stability of the GO–CoFe_2_O_4_–Silicone Magnetic Sponge over repeated cycles.

Figure 10 shows the variation in the water contact angle (WCA) of the GO–CoFe_2_O_4_–Silicone Magnetic Sponge as a function of repeated absorption–desorption cycles. The initial water contact angle was above 161.5°, confirming the superhydrophobic nature of the sponge surface. With increasing cycle number, a gradual decrease in the contact angle was observed; however, even after 20 cycles, the WCA remained higher than 145°, which is well above the threshold value of 150° commonly associated with highly hydrophobic surfaces.

The slight reduction in the water contact angle can be attributed to minor surface abrasion and partial loss of low-surface-energy components during repeated mechanical squeezing and reuse. Nevertheless, the sponge retained excellent water-repellent behavior throughout the entire cycling test, indicating that its hierarchical surface roughness and silicone-based hydrophobic coating remained largely intact.

These results demonstrate that the GO–CoFe_2_O_4_–Silicone Magnetic Sponge exhibits outstanding hydrophobic durability and structural stability under repeated operation conditions, which is crucial for its practical application in oil–water separation and wastewater treatment [24].

To further validate the effect of the composite modification, pristine PU sponge was used as a control sample in wettability and oil/water adsorption experiments (Figure S2). Visual droplet tests were performed by depositing both water and oil droplets onto the surfaces of unmodified and modified sponges. The pristine PU sponge showed rapid absorption of water, while oil droplets remained on its surface without significant uptake, indicating strong hydrophilicity but poor oleophilicity (Figure S2a). In contrast, the GO–CoFe_2_O_4_–silicone magnetic sponge exhibited a mirror-like surface after water deposition, with water droplets unable to penetrate, whereas oil droplets were quickly absorbed, demonstrating high oleophilicity and selective oil adsorption (Figure S2b). This stark contrast confirms that the enhanced selective adsorption performance is directly induced by the GO–CoFe_2_O_4_–silicone composite modification, highlighting the functional role of surface engineering, interfacial chemistry, and wettability control in achieving efficient oil capture and water repellency.

The absorption and selective separation performance of the GO–CoFe_2_O_4_–Silicone Magnetic Sponge was further evaluated through a continuous oil/water separation experiment using a vacuum-assisted filtration setup (Figure S3) [17,25]. The experimental system consisted of a vacuum pump, a filter flask, a rubber tube, and the GO–CoFe_2_O_4_–Silicone Magnetic Sponge positioned at the inlet of the suction tube. In a typical experiment, an oil/water mixture with a known composition was subjected to continuous suction. As shown in Figure S3a,b, the sponge selectively absorbed the oil phase upon contact with the mixture, while water was effectively excluded due to the superhydrophobic and oleophilic surface of the sponge. Under vacuum-driven suction, the absorbed oil was rapidly transported through the rubber tube and collected in the connected glass flask, forming a continuous oil stream without visible water contamination. After the separation process, the residual water phase became visibly clear, and no oil film was observed on the water surface (Figure S3c), indicating highly efficient oil removal. The separation efficiency (R, %) was calculated based on the mass difference of the oil phase before and after separation using Equation (2). The GO–CoFe_2_O_4_–Silicone Magnetic Sponge exhibited a separation efficiency exceeding 99%, demonstrating excellent selectivity and stability during continuous operation.

All separation experiments were conducted in triplicate, confirming the reproducibility and reliability of the vacuum-assisted continuous separation process. These results highlight the strong potential of the GO–CoFe_2_O_4_–Silicone Magnetic Sponge for practical applications in oil/water separation and wastewater treatment under dynamic flow conditions.

As summarized in Table 1, our GO–CoFe_2_O_4_–Silicone Magnetic Composite demonstrates absorption capacities and separation efficiencies that are comparable or superior to recently reported magnetic graphene-based composites for oil/water separation. Notably, membranes based on Fe_3_O_4_/RGO and fluorinated GO composites have shown high efficiencies, but often with lower reuse potential or under different testing conditions. In contrast, our composite combines high adsorption capacity, >99% separation efficiency, and robust cyclic stability, highlighting its practical potential.

The proposed composite system is based on low-cost, commercially available, and scalable components, including PU sponge substrates, graphene oxide, cobalt ferrite precursors, and silicone oil. The fabrication process relies on solution processing, impregnation, and thermal treatment, without the need for expensive equipment or complex synthesis routes, making it industrially feasible. Moreover, the magnetic recyclability and reusability of the composite significantly reduce operational costs [29]. These features highlight the strong potential of the material for practical large-scale oil–water separation and wastewater treatment applications.

## 4. Conclusions

In this work, a multifunctional GO–CoFe_2_O_4_–silicone magnetic sponge was successfully fabricated as an efficient material for oil and organic solvent removal from water. The integration of graphene oxide and CoFe_2_O_4_ nanoparticles with silicone surface modification provided the sponge with a unique combination of superhydrophobicity, oleophilicity, and magnetic responsiveness. The modified sponge exhibited a high water contact angle of 161.5°, confirming its excellent water-repellent behavior, while the presence of the magnetic ferrite phase enabled convenient magnetic manipulation and recovery. The developed material demonstrated high adsorption capacities (21–45 g g^−1^) toward a variety of oils and organic solvents, together with separation efficiencies exceeding 99% for different oil/water systems. Furthermore, the sponge retained its adsorption performance over repeated adsorption–desorption cycles, indicating good structural stability and reusability. The synergistic combination of the porous polyurethane scaffold, hierarchical surface roughness, and low-surface-energy silicone coating plays a key role in achieving efficient selective oil uptake and water repellency. Overall, the GO–CoFe_2_O_4_–silicone magnetic sponge represents a robust, reusable, and magnetically recoverable material with strong potential for practical oil–water separation and wastewater remediation. Owing to its simple fabrication procedure and the use of readily available components, the proposed system may offer a promising platform for the development of scalable materials for environmental cleanup applications.

## 5. Patents

Sugurbekova, G.K.; Kudaibergenova, R.M.; Ualibek, O.; Sugurbekov, Y.T.; Demeuova, G.K. Method of producing an oil-absorbing magnetic sponge. Patent for Utility Model. №8437, 22.09.2023.

## Figures and Tables

**Table 1:** Comparison of GO–CoFe_2_O_4_–Silicone Magnetic Composite with reported magnetic graphene-based materials for oil/water separation.

Material/Composite	Composition/Structure	Adsorption Capacity (g/g)	Separation Efficiency (%)	Reusability/Cycles	Reference
GO–CoFe_2_O_4_–Silicone Magnetic Composite (this work)	GO + CoFe_2_O_4_ + Silicone	Chloroform: 44.83 g/g; Hexane: 21.38 g/g; others in between	>99% (oil/water mixtures)	5 cycles, minimal loss	Current study
Fe_3_O_4_/RGO/SA composite membrane	Fe_3_O_4_ + RGO + stearic acid	not reported	>98.4%	>10 cycles	[26]
Fe_3_O_4_@GO@Ka (magnetic clay composite)	Fe_3_O_4_ + GO + kaolin	not reported	High, effective for emulsions	Multi-cycle, stable	[27]
Fluorinated-polyether grafted GO–Fe_3_O_4_	Fe_3_O_4_ + GO + fluoropolymer	not reported	~91.4% demulsification	4 cycles, efficiency > 70%	[25]
Superhydrophobic magnetic ZS@BIF composite	Mineral-based magnetic hydrophobic composite	~22 g/g (cyclohexane)	High	Comparable to other composites	[28]
CNT-based magnetic composite (PU/CNT/NiFe_2_O_4_/PDMS)	CNT + NiFe_2_O_4_ + PDMS	21–45 g/g	>99%	5 cycles, stable	[25]

## Data Availability

All data supporting the findings of this study are included in the article and the Supplementary Materials. Additional raw data, including XRD, FTIR, VSM, contact angle measurements, adsorption capacity datasets, and kinetic/separation experiments, are available from the corresponding author upon reasonable request.

## Supplementary Materials

The following supporting information can be downloaded at: https://www.mdpi.com/article/10.3390/nano16060345/s1, Video S1: Demonstration of magnetic attraction of the GO–CoFe_2_O_4_–Silicone Magnetic Sponge; Figure S1: Photographs illustrating the removal of crude oil from an oil/water mixture using the GO–CoFe_2_O_3_–silicone sponge; Figure S2: Continuous oil/water separation performance of the GO–CoFe_2_O_3_–Silicone Magnetic Sponge under vacuum-assisted filtration: (a) before separation; (b) during separation; and (c) after separation; Figure S3: Continuous oil/water separation performance of the GO–CoFe_2_O4–Silicone Magnetic Sponge under vacuum-assisted filtration: (a) before separation; (b) during separation; and (c) after separation.
