# Supplementary material for: Interface-Controlled GO–CoFe2O4–Silicone Nanocomposite with Magnetic and Adsorptive Functionality
Source: Nanomaterials (Basel). 2026 Mar 11;16(6):345. doi: 10.3390/nano16060345 (PMC13029722; doi:10.3390/nano16060345)
Supplement: Supplementary file 1 [file nanomaterials-16-00345-s001.zip › nanomaterials-4163691-supplementary.pdf]

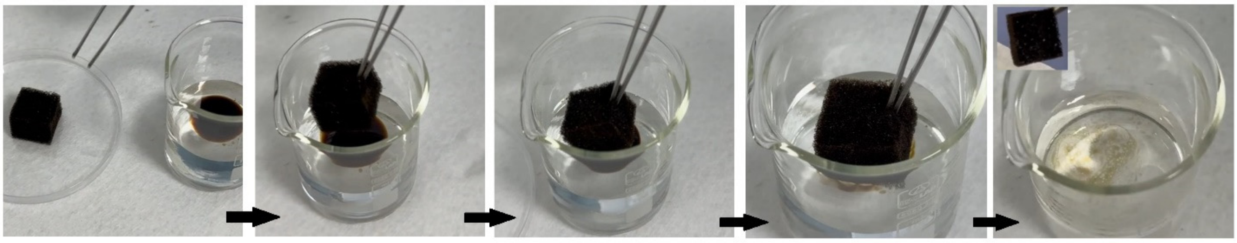

Figure S1. Photographs illustrating the removal of crude oil from an oil/water mixture using the GO–$CoFe_2O_4$–silicone sponge.

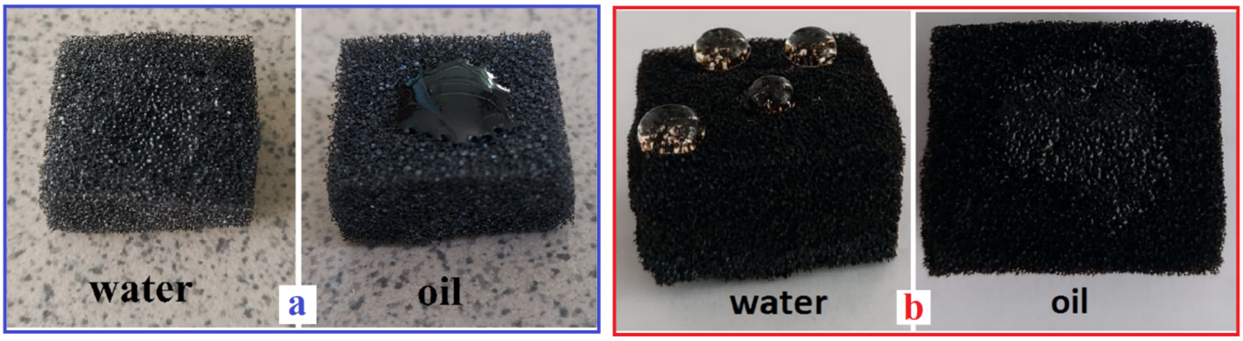

Figure S2. Comparative water/oil droplet adsorption behavior on (a) pristine PU sponge and (b) GO–$CoFe_2O_4$–silicone magnetic sponge

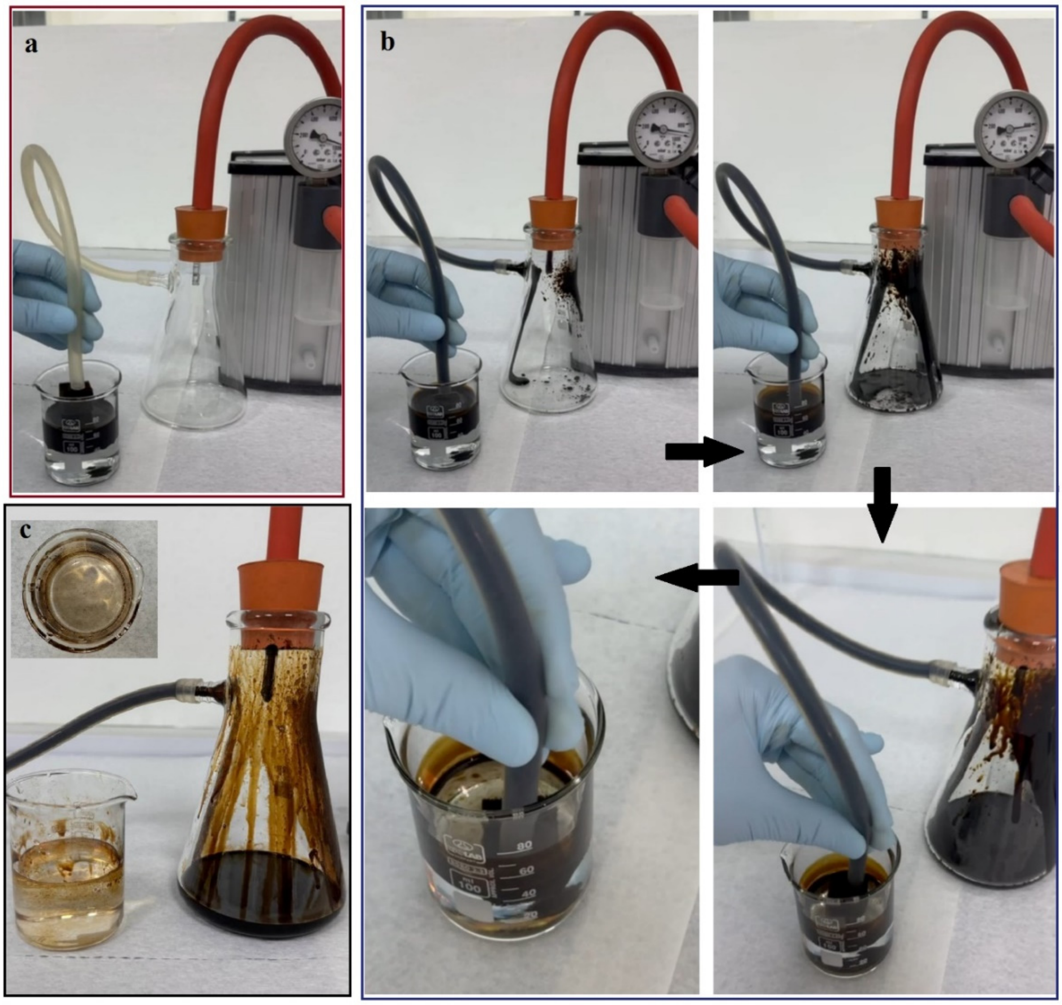

Figure S3. Continuous oil/water separation performance of the GO–$CoFe_2O_4$–Silicone Magnetic Sponge under vacuum-assisted filtration: (a) before separation; (b) during separation; and (c) after separation.
